# Decapsulation of the Kidney in Nephritis

**Published:** 1904-04-16

**Authors:** 


### DECAPSULATION OF THE KIDNEY IN NEPHRITIS.

It is not altogether unnatural that the proposal to treat chronic nephritis by such a bold undertaking as decapsulation of the kidney should meet with a good deal of adverse criticism. But mere theoretical arguments are of small worth compared with practical observation, and accordingly extensive experiments have been carried out recently on animals with a view to settling the matter. It will be remembered that when Edebohls first thought of treating chronic nephritis by stripping off the capsule of the kidney his idea was based upon observation of the benefits which followed the fixation of a movable kidney by a method which involved a partial decapsulation. Further, in a case which died four months after decapsulation he thought he observed enormously dilated and enlarged blood-vessels which penetrated from the fatty capsule through the capsule proper into the kidney substance. This increased supply of blood to the damaged kidney, he believed, would bring about regenerative changes and facilitate excretion. It is clear that if an increased blood supply does occur after removal of the capsule there is a fair prospect of a damaged kidney benefiting thereby, but if no appreciable development of new blood-vessels occurs, the premises from which Dr. Edebohls sets out are at fault, and his theory, if not his practice, is wrong. To put the matter to a test, Dr. J. Walker Hall and Dr. G. Herxheimer[1] have carried out some experimental work, the results of which they have reported to the Scientific Grants Committee of the British Medical Association. The main object of their inquiry was to investigate the tissue changes which follow the decapsulation of kidneys in which nephritis has been previously induced. They state that their observations on the effects of the operation in animals with healthy kidneys agree with those of other experimenters. They find that when the capsule is removed from a healthy kidney it reforms early, and at the end of 10 to 21 days is represented by a fibrous covering thicker than the original capsule. After decapsulation of the kidney in 35 rabbits they were not able to find, either by ordinary or by serial sections, any marked formation of new blood channels between the kidney and the adherent tissues. They then directed their attention to the production of acute degenerative nephritis and to subsequent decapsulation of the kidney in order to approach more nearly the conditions under which the operation of Edebohls has its practical application. The nephritis was produced by injecting rabbits with a dose of 0·5 to 0·75 c.cm. of a 2·5 per cent. solution of neutral ammonium chromate. This produced a very intense acute congestion, a necrosis of the cells of the convoluted tubules, and the appearance of albumen and casts in the urine within 24 hours. As a rule nephritis was first induced and decapsulation performed three days later, but in a few cases the procedure was reversed. In either case the results were practically the same. The stripped off capsule was soon replaced by a thicker and denser one, and no new formation of blood-vessels passing from the surrounding tissues into the kidney substance could be observed. It may be fairly concluded, therefore, that as there apparently exists no anatomical basis for the operation in acute degenerative nephritis the same is true of chronic nephritis. Nor can the improvement noticed clinically after decapsulation in man be attributed to the relief of congestion, for the authors found that the intense hyperæmia persisted after the capsule had reformed. They conclude that in view of the above facts the theory upon which is based the practice of renal decapsulation is wrong, and that such benefits as may accrue to the patient by the procedure are likely to be obtainable by milder methods such as reni-puncture.

[1] Brit. Med. Jour., April 9.

